# Transition-Metal-Free Activation of Amide Bond by Arynes

**DOI:** 10.3390/molecules23092145

**Published:** 2018-08-27

**Authors:** Hideto Miyabe

**Affiliations:** School of Pharmacy, Hyogo University of Health Sciences, Minatojima 1-3-6, Chuo-ku, Kobe 650-8530, Japan; miyabe@huhs.ac.jp; Tel.: +81-78-304-3094

**Keywords:** amide, arynes, insertion, activation, heterocycles, organic synthesis, multi-component coupling reaction

## Abstract

Highly reactive arynes activate the N–C and C=O bonds of amide groups under transition metal-free conditions. This review highlights the insertion of arynes into the N–C and C=O bonds of the amide group. The insertion of arynes into the N–C bond gives the unstable four-membered ring intermediates, which are easily converted into *ortho*-disubstituted arenes. On the other hand, the selective insertion of arynes into the C=O bond is observed when the sterically less-hindered formamides are employed to give a reactive transient intermediate. Therefore, the trapping reactions of transient intermediates with a variety of reactants lead to the formation of oxygen atom-containing heterocycles. As relative functional groups are activated, the reactions of arynes with sulfinamides, phosphoryl amides, cyanamides, sulfonamides, thioureas, and vinylogous amides are also summarized.

## 1. Introduction

**Scheme 1:**
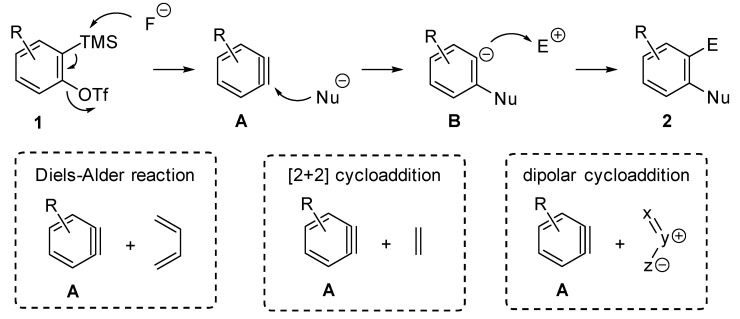
Transition metal-free reaction of arynes.

In recent years, the use of arynes as highly reactive and strained intermediates in organic synthesis has attracted substantial attention [1,2,3,4,5,6,7,8,9,10,11,12,13,14,15]. Arynes have been extensively utilized in transition-metal-catalyzed reactions [16,17]. The development of *ortho*-trimethylsilyl aryltriflates **1** as mild aryne precursors led to growing activity in this field (Scheme 1) [18]. Arynes **A** can be generated in situ from triflate **1** and fluoride ion under mild reaction conditions. Therefore, the aryne chemistry using aryltriflates **1** has achieved some remarkable success, particularly in the transition metal-free reactions.

Most of transition metal-free reactions proceed through the addition of nucleophiles to arynes **A** and the subsequent trapping of intermediates **B** with electrophiles to give multi-substituted arenes with structural diversity and complexity. The transition metal-free concerted reactions, such as the Diels-Alder reaction, [2 + 2] cycloaddition reaction, and dipolar cycloaddition reaction, are also synthetically useful [6,7,11,12].

**Scheme 2:**
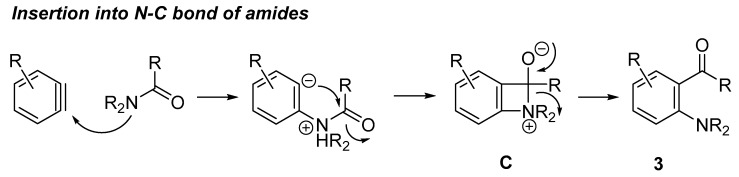
Activation of amide N–C bond by arynes.

**Scheme 3:**
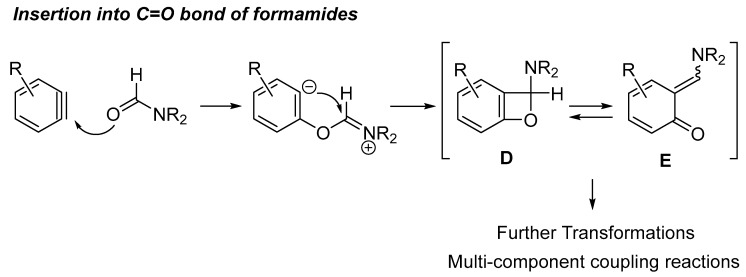
Activation of amide C=O bond by arynes.

When the nitrogen atom of amides acts as nucleophiles toward arynes, the insertion of arynes into the N–C bond is induced to give the N–C insertion products **3**, via the formation of four-membered ring intermediates, **C** (Scheme 2). In contrast, insertion into the C=O bond is promoted by the nucleophilic addition of the oxygen atom of amides to arynes (Scheme 3). In the C=O insertion reaction, the four-membered ring intermediates **D** and *ortho*-quinone methides **E** are highly reactive [19,20]; thus, a variety of further transformations using **D** or **E** have been developed as multi-component coupling reactions [9]. As shown in Section 3 with the C=O bond activation, the suitable amides for C=O insertion are the sterically less-hindered formamides, such as *N*,*N*-dimethylformamide (DMF).

## 2. N–C Bond Activation

**Scheme 4:**
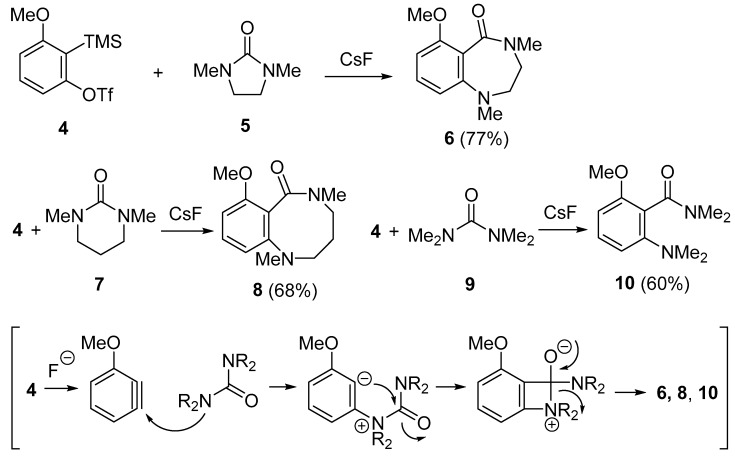
Reaction of ureas with aryne.

At first, the insertion of arynes into the N–C bond of the amide group was reported in the reaction of ureas with arynes [21]. In the presence of CsF, treatment of 3-methoxy-2-(trimethylsilyl) phenyl triflate **4** as an aryne precursor with 1,3-dimethyl-2-imidazolidinone (DMI) **5** gave 1,4-benzodiazepine derivative **6** in 77% yield (Scheme 4). Under similar reaction conditions, *N*,*N*′-dimethylpropyleneurea (DMPU) **7** worked well to give 1,5-benzodiazocine derivative **8**. The insertion of aryne into the N–C bond of acyclic *N*,*N*,*N*′,*N*′-tetramethylurea **9** also proceeded. In these reactions, aryne is generated by the reaction of triflate **4** with the fluoride anion of CsF. The sequential transformation is achieved via a route involving the addition of the urea nitrogen atom to an aryne, followed by the intramolecular nucleophilic attack on the carbonyl carbon atom. The resulting four-membered ring intermediate readily undergoes ring opening to afford the N–C insertion products **6**, **8**, and **10**.

**Scheme 5:**
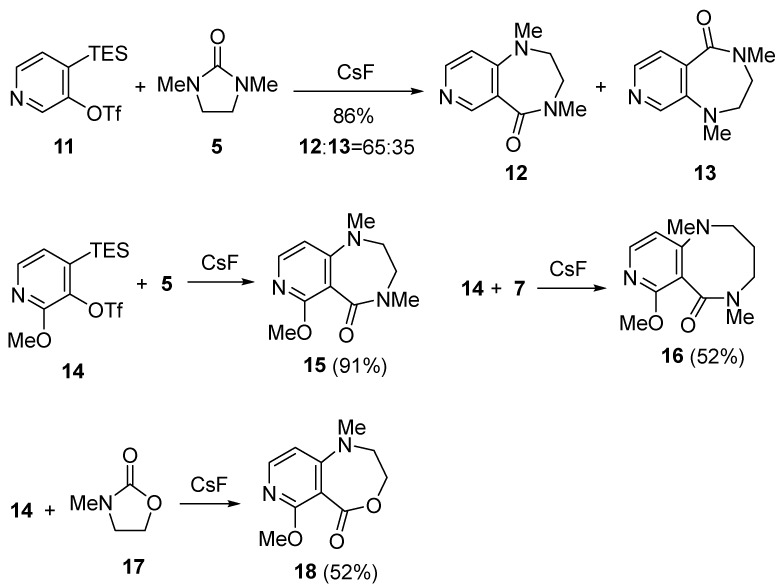
Insertion of pyridynes into N–C bond.

The reaction of pyridynes with ureas was studied [22]. In the presence of CsF, the reaction of 4-triethylsilyl-3-trifluoromethanesulfonyloxypyridine **11** as a 3,4-pyridyne precursor with DMI **5** gave pyridodiazepine derivatives **12** and **13** in 86% yield and a ratio of 65:35 (Scheme 5). High regioselectivity was obtained by using the 3,4-pyridyne precursor **14** having a methoxy group at the 2-position to give the product **15**, selectively. The use of DMPU **7** instead of DMI **5** led to the formation of the corresponding pyridodiazocine, **16**. When 1-methyl-2-oxazolidone **17** was employed, the selective insertion into the N–C bond of **17** proceeded to give pyridooxazepine **18**.

**Scheme 6:**
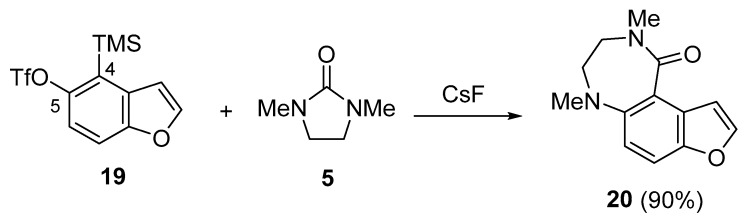
Insertion of 4,5-benzofuranyne into N–C bond.

The reaction of DMI **5** with 4,5-benzofuranyne precursor **19** was also studied (Scheme 6) [23]. The N–C insertion product **20** was regioselectively obtained in 90% yield as a result of the initial attack of DMI **5** at C5 of 4,5-benzofuranyne.

**Scheme 7:**
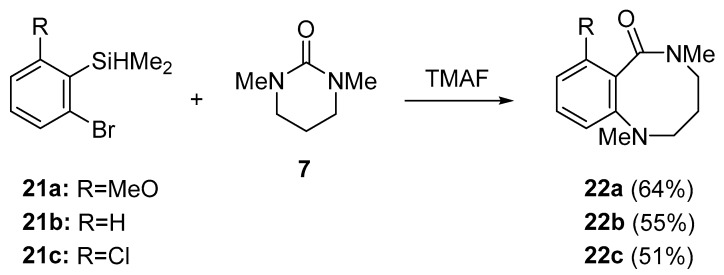
Reaction of silylaryl bromides **21a**–**c** with DMPU **7**.

It is reported that silylaryl bromides and iodides can be used as aryne precursors under the conditions similar to those employed for silylaryl triflates, such as precursors **4**, **11,** and **19** [24]. The utility of silylaryl bromides **21a**–**c** was demonstrated in the N–C bond reaction (Scheme 7). In the presence of tetramethylammonium fluoride (TMAF), 1-bromo-3-methoxy-2-(dimethylsilyl) benzene **21a** reacted with DMPU **7** to give **22a** in 64% yield. Silylaryl bromides **21b** and **21c** also worked well.

**Scheme 8:**
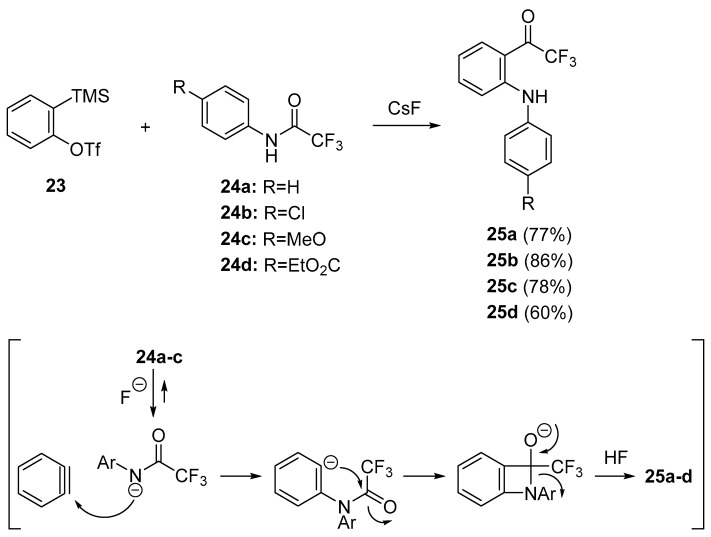
Reaction of *N*-phenyltrifluoroacetamides with aryne.

The insertion of arynes into the N–C bond of *N*-phenyltrifluoroacetamides proceeded effectively [25]. In the presence of CsF, the reaction of *N*-phenyltrifluoroacetamide **24a** with triflate **23** as an aryne precursor gave the N–C insertion product **25a** in 77% yield (Scheme 8). The substituted *N*-aryltrifluoroacetamides **24b**–**d** also afforded the corresponding products **25b**–**d** in good yields. Since the CF_3_ group on amides is critical to the success of these transformations, they propose the reaction mechanism involving the abstraction of the hydrogen on amide nitrogen by fluoride anion as a base. The products **25a**–**d** are obtained via the attack of amide nitrogen anion to aryne, the intramolecular trapping process with the carbonyl carbon atom, and the four-membered ring opening.

**Scheme 9:**
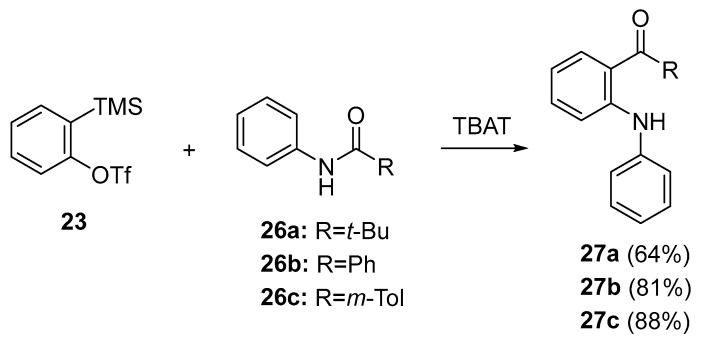
Reaction of *N*-phenyltrifluoroacetamides with aryne.

To develop the amide insertion reaction having broad utility, the reaction of *N*-pivaloylaniline **26a** with triflate **23** was investigated by changing solvents and fluoride sources [26]. Employing tetrabutylammonium triphenyldifluorosilicate (TBTA) as a fluoride source, amide **26a** underwent the N–C insertion in toluene at 50 °C to afford the *tert*-butylketone **27a** in 64% yield (Scheme 9). Exploration of substrate scope showed that *N*-phenyl derivatives **26b** and **26c** were similarly efficient substrates.

**Scheme 10:**
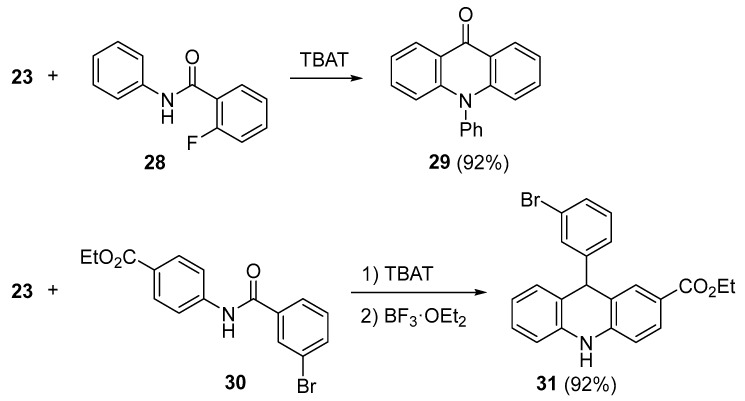
Synthesis of acridone and acridine.

Additionally, this reaction was applied to the synthesis of acridones and acridines (Scheme 10). The one-step synthesis of acridone **29** was achieved by the reaction of *ortho*-halobenzamide **28**, with triflate **23** under microwave irradiation at 120 °C in the presence of TBAT. Acridone **29** was formed via a route involving the N–C insertion, followed by the intramolecular S_N_Ar reaction. In contrast, acridine **31** was synthesized by a one-pot procedure using BF_3_·OEt_2_ via a route involving the N–C insertion of amide **30** into aryne, followed by a BF_3_-mediated Friedel-Crafts acylation and dehydration.

**Scheme 11:**
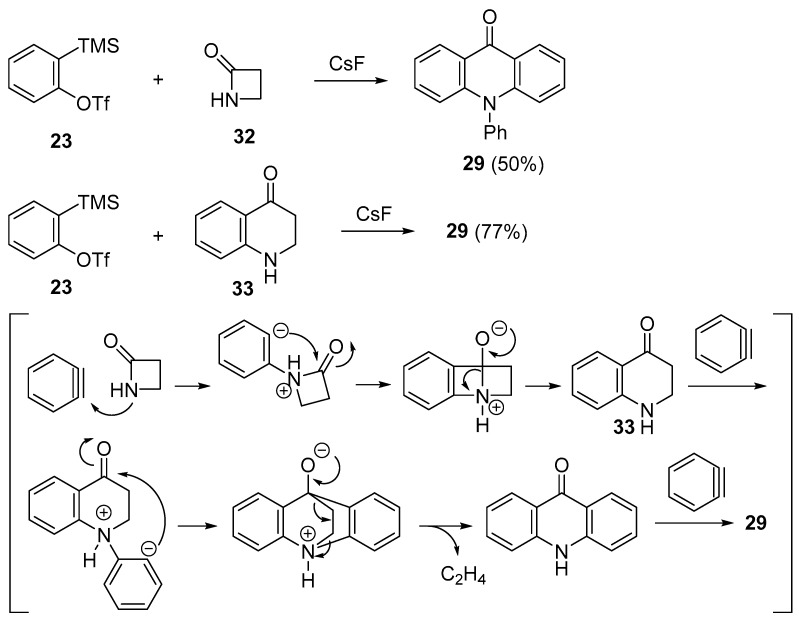
Reaction of β-lactam with aryne leading to acridone.

The reaction of β-lactam **32** with aryne gave acridone **29** in 50% yield by employing 3.5 equivalents of the aryne precursor **23** in the presence of CsF (Scheme 11) [27]. In this transformation, 2,3-dihydroquinolin-4-one **33** is formed as an intermediate as a result of N–C bond insertion of aryne into β-lactam **32**. In fact, **33** reacted under the same reaction conditions to give acridone **29** in 77% yield. The conversion of **33** into **29** will proceed through the *N*-arylation of **33** with second aryne, the subsequent cyclization, the extrusion of ethylene, and the final *N*-arylation with third aryne.

**Scheme 12:**
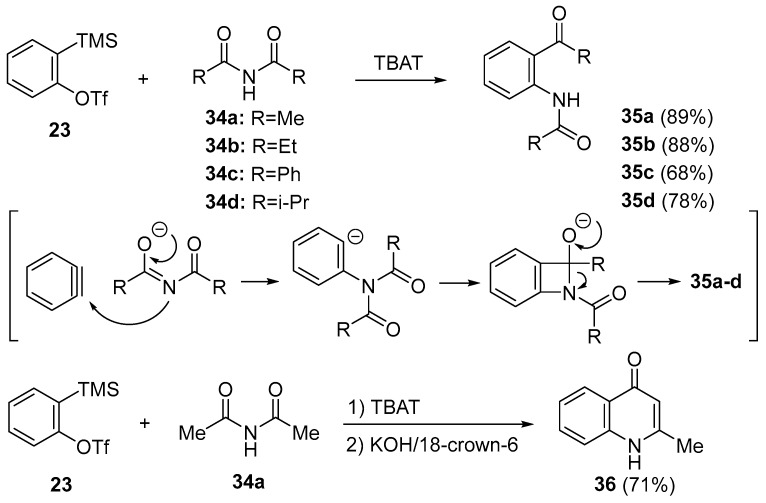
Reaction of imides with aryne.

The insertion of arynes into the N–C bond of imides was investigated [28]. The formation of simple *N*-arylated products could be suppressed when the reactions of imides **34a**–**d** with triflate **23** were carried out in toluene at 60 °C in the presence of TBAT (Scheme 12). The desired N–C insertion products **35a**–**d** were selectively obtained. Additionally, this reaction was applied to the one-pot synthesis of quinolone **36** through Camps cyclization using KOH and 18-crown-6.

## 3. C=O Bond Activation

**Scheme 13:**
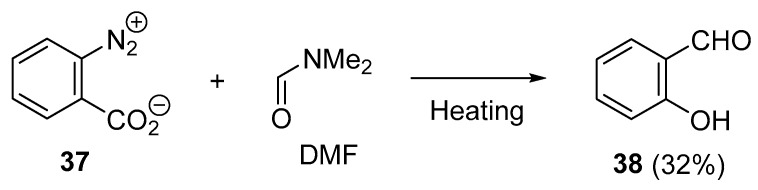
Reaction of *N*,*N*-dimethylformamide with aryne.

At first, the insertion of arynes into the C=O bond of the amide group was reported [29]. Aryne, generated from precursor **37**, reacted with *N*,*N*-dimethylformamide (DMF) to give salicylaldehyde **38** in 32% yield (Scheme 13).

**Scheme 14:**
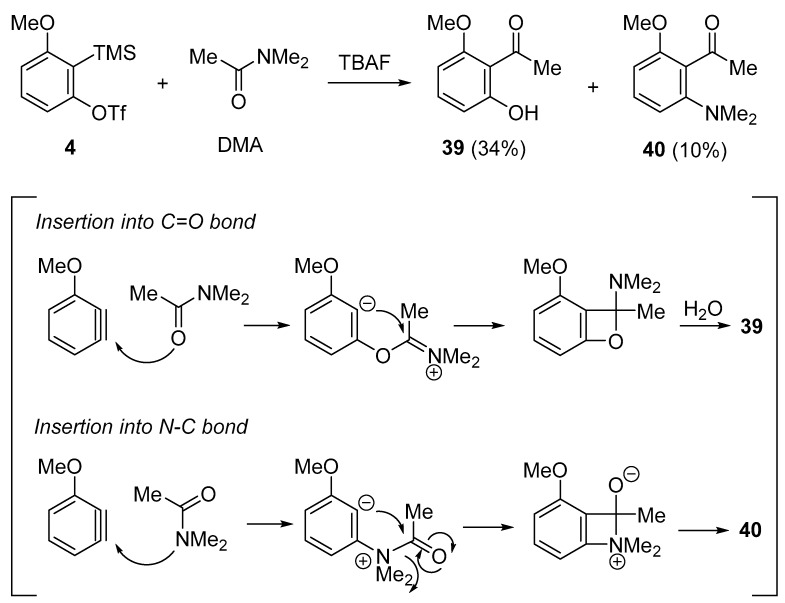
Reaction of *N*,*N*-dimethylacetamide with aryne.

When the bulky *N*,*N*-dimethylacetamide (DMA) was used, competitive insertion into the C=O and N–C bonds of DMA was observed [30]. In the presence of TBAF, treatment of **4** with DMA gave the C=O insertion product **39** in 34% yield, and the N–C insertion product **40** in 10% yield (Scheme 14). This result indicates that the sterically less-hindered formamides are the suitable nucleophiles for C=O insertion. The insertion into the C=O bond will proceed via the stepwise mechanism involving the addition of the oxygen atom of amide to an aryne, followed by the intramolecular nucleophilic attack on the iminium.

**Scheme 15:**
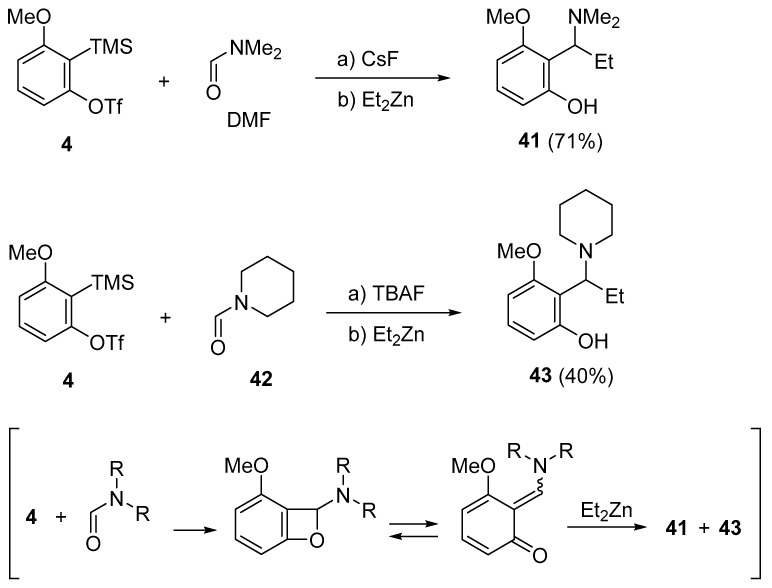
Reaction for trapping the transient intermediates.

The sequential reaction involving the trapping process of transient intermediates with organometallic reagents was studied [30,31]. After a solution of triflate **4** in DMF was stirred in the presence of CsF, a solution of Et_2_Zn in hexane was added to the reaction mixture (Scheme 15). The desired aminophenol **41** was obtained in 71% yield. Diethyllzinc also trapped the transient intermediate generated from triflate **4** and formamide **42,** to give the aminophenol **43** by a one-pot procedure.

**Scheme 16:**
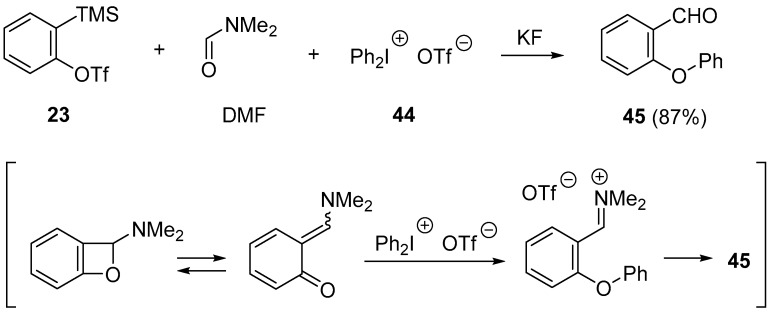
Trapping reaction using diphenyliodonium salt.

Three-component sequential coupling of arynes, DMF, and diaryliodonium salts was studied [32]. In the presence of KF, a three-component coupling reaction was found using triflate **23** and diphenyliodonium triflate **44** in DMF-facilitated 2-phenoxybenzaldehyde **45** in 87% yield (Scheme 16). In this transformation, diphenyliodonium triflate **44** acted as an electrophile by trapping the oxygen atom of a transient intermediate.

**Scheme 17:**
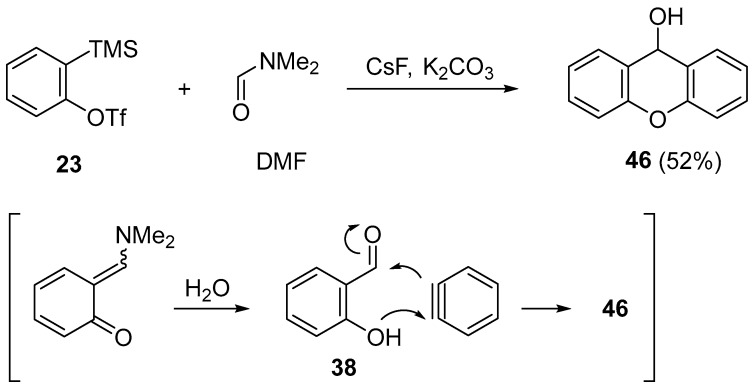
2:1-Coupling reaction.

The 2:1 coupling reaction of two molar amounts of aryne and one molar amount of DMF was reported (Scheme 17) [33]. Initially, the reaction of precursor **23** and DMF gives salicylaldehyde **38** via the hydrolysis of a transient intermediate. 9-Hydroxyxanthene **46** is formed by the reaction of salicylaldehyde **38** with aryne.

**Scheme 18:**
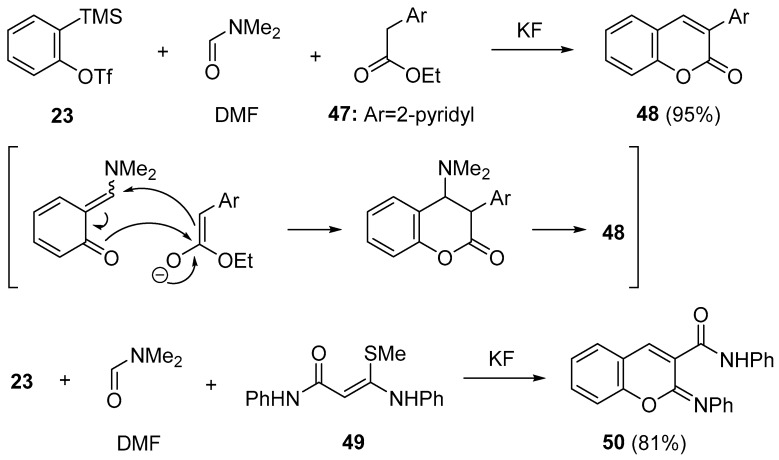
Synthesis of coumarin derivatives.

The trapping reactions of transient intermediates generated from arynes precursors and DMF with a variety of reactants have been widely studied as being synthetic approaches to oxygen atom-containing heterocycles [34,35,36,37,38,39,40,41,42,43]. The synthesis of 2*H*-coumarin derivatives was also studied [34,35,36]. Three-component coupling reactions leading to chromene **48** was achieved by the use of acetate **47,** having an aryl group as a nucleophile for trapping the unstable intermediate (Scheme 18). In the presence of KF, the reaction of triflate **23** and acetate **47** was carried out in DMF at 80 °C to give the coumarin **48** in 95% yield [35]. The synthesis of 2-aryliminochromene skeleton of biologically active compounds was studied by using a three-component coupling reaction [36]. A transient intermediate, generated from triflate **23** and DMF, could be trapped by *N*,*S*-keteneacetal **49** to give the biologically important arylimino-2*H*-chromene-3-carboxamide **50** in 81% yield. The synthesis of 4*H*-chromene derivatives was also achieved by using a three-component coupling reaction involving the hetero Diels-Alder reaction between transient intermediates and dienophiles [37].

**Scheme 19:**
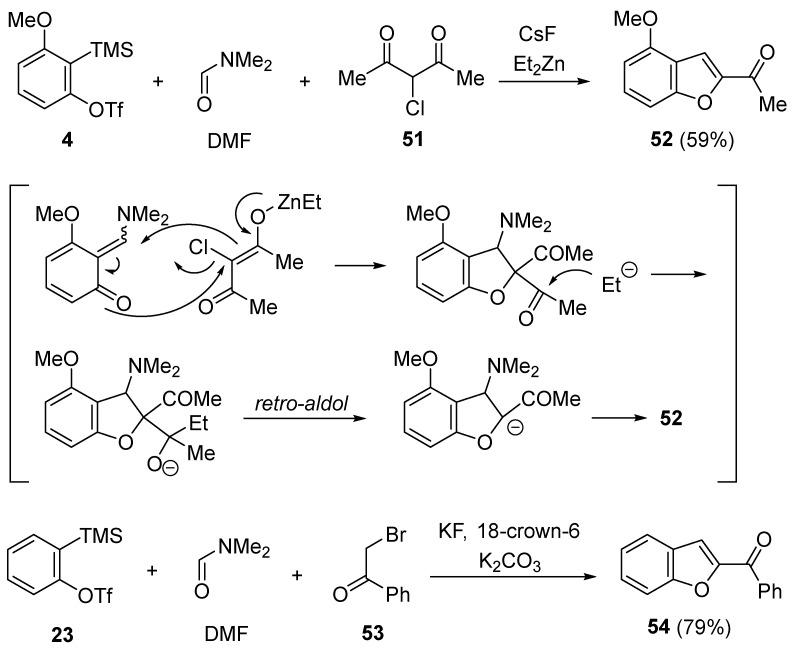
Synthesis of benzofurans.

The synthesis of benzofurans was also studied [38,39,40]. The use of α-halogenated enolate, generated from α-chloromalonate **51** and Et_2_Zn, led to the formation of benzofuran **52** (Scheme 19) [38]. In the presence of CsF, treatment of aryne precursor **4** and α-chloromalonate **51** with Et_2_Zn in DMF gave **52** in 59% yield. In this transformation, α-chloromalonate acts as a nucleophilic and electrophilic one carbon-unit for trapping a transient intermediate. Benzofuran **52** will be formed via a route involving the retro-aldol type reaction. The simple one-pot synthesis of benzofurans was also reported [40]. When 2-bromoacetophenone **53** was used as a nucleophilic and electrophilic reactant, benzofuran **54** was obtained in 79% yield.

Additionally, the trapping reaction of transient intermediates was successfully applied to a four-component coupling reaction for the convenient synthesis of xanthene derivatives [34,41,42].

## 4. Activation of Relative Bonds

**Scheme 20:**
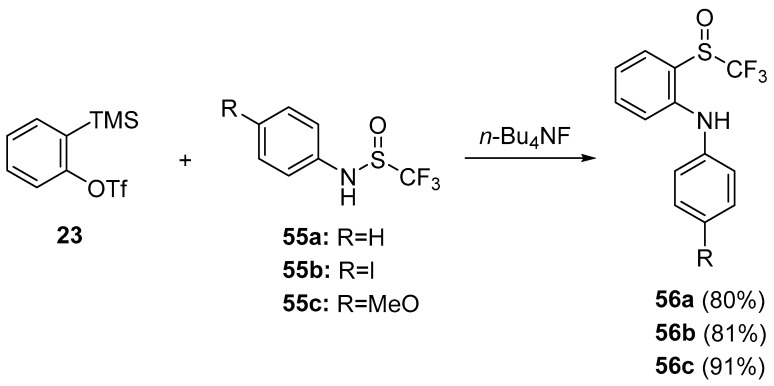
Reaction of *N*-phenyltrifluoromethanesulfinamides with aryne.

The insertion of arynes into the N–S bond of sulfinamides was studied [25]. In the presence of *n*-Bu_4_NF, the reaction of *N*-phenyltrifluoromethanesulfinamides **55a**–**c** with triflate **23** as an aryne precursor gave the corresponding N–S insertion products **56a**–**c** in good yields (Scheme 20).

**Scheme 21:**
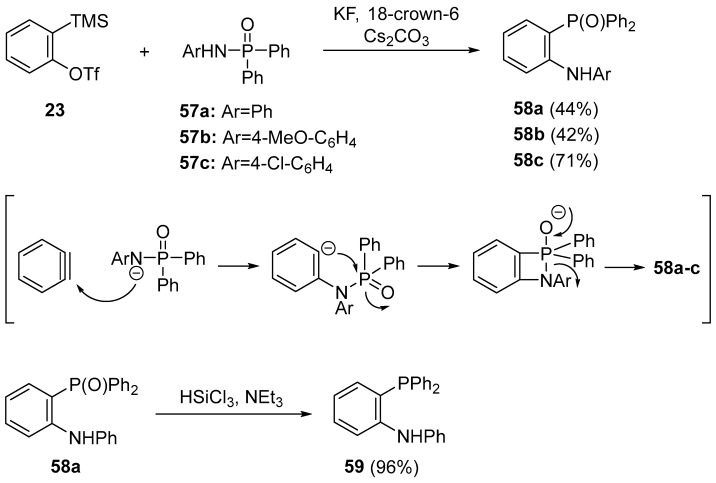
Reaction of diphenylphosphinic amides with aryne.

The insertion of arynes into the P–N bonds of arylphosphoryl amides was studied [44]. In the presence of KF and 18-crown-6, the reaction of diphenylphosphinic amides **57a**–**c** with triflate **23** was carried out at 80 °C in a sealed tube (Scheme 21). The *ortho*-aniline-substituted arylphosphine oxides **58a**–**c** were obtained in moderate yields. This transformation proceeded through the addition of the nitrogen atom of **57a**–**c** to an aryne, the intramolecular trapping, and the four-membered ring opening. Additionally, the P–N insertion product **58a** was converted to *ortho*-amine-substituted arylphosphine **59** in 96% yield by the reduction using HSiCl_3_.

**Scheme 22:**
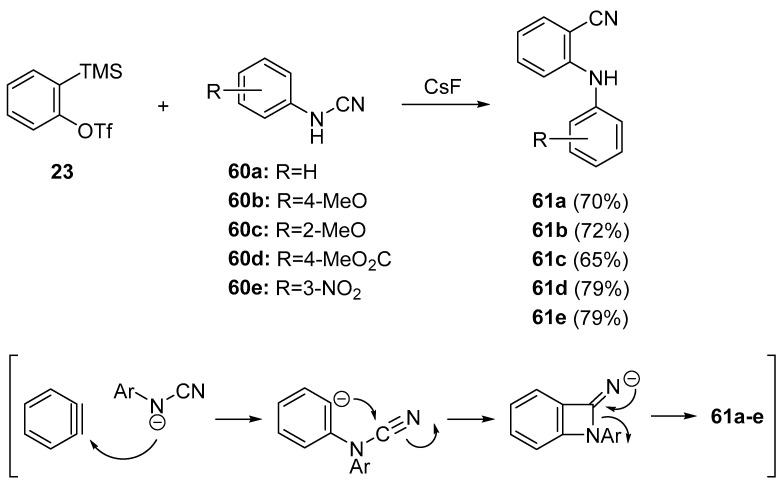
Reaction of aryl cyanamides with aryne.

The insertion of arynes into the N–C bonds of aryl cyanamides was reported [45]. In the presence of CsF, triflate **23** reacted with aryl cyanamides **60a**–**e** to give the 1,2-bifunctional aminobenzonitriles **61a**–**e** in good yields (Scheme 22). This N–C bond insertion also proceeds via the formation of the four-membered ring intermediates.

**Scheme 23:**
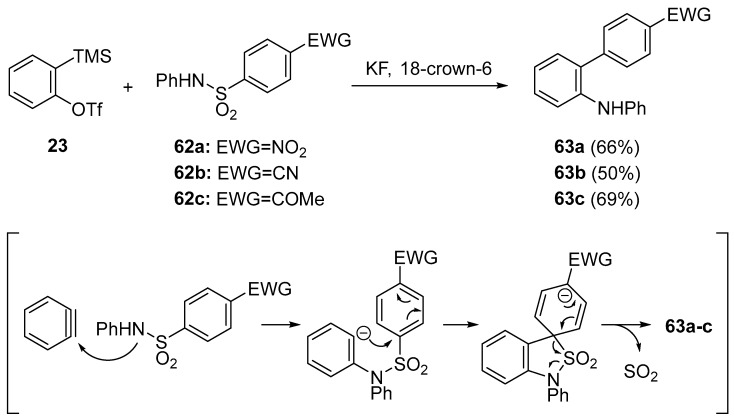
Reaction of aryl sulfonamides with aryne.

The synthesis of biaryl compounds was achieved by using the reaction of aryl sulfonamides with arynes [46]. In the presence of KF and 18-crown-6, aryl sulfonamides **62a**–**c** having an electron-withdrawing group reacted with aryne to afford 2-amino-biaryls **63a**–**c** (Scheme 23). This reaction involves the addition of sulfonamides to aryne, and the subsequent Smiles-type *ipso*-substitution with sulfur dioxide SO_2_ extrusion.

**Scheme 24:**
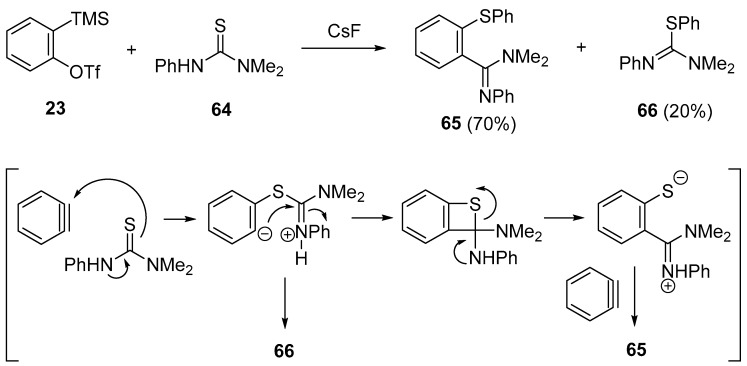
Reaction of thiourea with aryne.

Formal π-insertion into the C=S bond was observed in the reaction of thioureas with aryne [47]. When a solution of triflate **23** and thiourea **64** in toluene/MeCN was heated in the presence of CsF, amidine **65** was formed in 70% yield, accompanied with the simple *S*-arylated product **66** in 20% yield (Scheme 24). The sequential transformation leading to **65** was started by the reaction of the sulfur atom of **64** with an aryne, which was followed by intramolecular trapping to give a four-membered ring intermediate. The amidine **66** was obtained via the four-membered ring opening and subsequent *S*-arylation by an aryne.

**Scheme 25:**
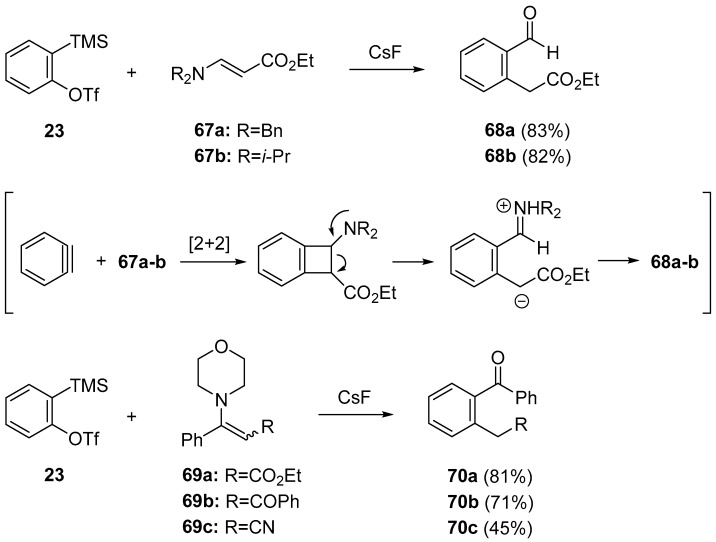
Reaction of vinylogous amides with aryne.

The C=C double bond of vinylogous amide derivatives reacted with aryne [48,49]. In the presence of CsF, the reaction of vinylogous amide derivatives **67a**–**b** with aryne gave the carbonyl compounds **68a**–**b** in good yields (Scheme 25). This transformation proceeded via the [2 + 2] cycloaddition between aryne and **67a**–**b** and the four-membered ring opening. The bulky vinylogous amides **69a**–**c** having ester, ketone, or cyano group as an electron-withdrawing group reacted well with aryne to give the corresponding products **70a**–**c** in good yields.

## 5. Concluding Remarks

Arynes are highly reactive intermediates that can activate the N–C and C=O bonds of an amide group under transition-metal-free conditions. As described above, the insertion of arynes into the N–C bond has been studied as a powerful method for preparing *ortho*-disubstituted arenes. In contrast, the selective insertion of arynes into the C=O bond proceeds when sterically less-hindered formamides are employed. Moreover, the trapping reactions of transient intermediates with a variety of reactants, leading to the multi-component coupling reaction, disclosed a broader aspect of the utility of N–C bond insertion for the synthesis of oxygen atom-containing heterocycle**s**. I hope that this review will inspire new creative contributions to organic chemists.
